# Emergence of extrathoracic manifestations of eosinophilic granulomatosis with polyangiitis during benralizumab treatment

**DOI:** 10.1093/rap/rkab033

**Published:** 2021-05-01

**Authors:** Andy K H Lim, Anna Antony, Michael Gingold, Ian Simpson, Wenye F Looi, Martin I MacDonald

**Affiliations:** 1 General Medicine, Monash Health; 2 Department of Medicine, School of Clinical Sciences, Monash University; 3 Rheumatology; 4 Anatomical Pathology; 5 Respiratory and Sleep Medicine, Monash Health, Clayton, Victoria, Australia

Key message

- Emergent eosinophilic granulomatosis with polyangiitis can occur with benralizumab (anti-IL-5 receptor) treatment despite depletion of blood eosinophils.

Dear Editor, We report on a 31-year-old man with emergence of eosinophilic granulomatosis with polyangiitis (EGPA) despite depletion of blood eosinophils by benralizumab (anti-IL-5 receptor). Seven months earlier, the patient was hospitalized with severe asthma. Chest CT demonstrated peribronchial wall thickening and ground-glass opacities. Total immunoglobulin E (IgE) and blood eosinophils were elevated at 4150 kU/l and 4.0 × 10^9^/l, respectively. Aspergillus serology was negative. Nasoendoscopy and bronchoscopy were unremarkable. Serum ANCA was negative. He was treated for severe eosinophilic asthma with prednisolone and, subsequently, benralizumab. Clinical stability was achieved, with improvements in his forced expiratory volume in 1 s and depletion of blood eosinophils. However, fractional exhaled nitric oxide (FeNO), a biomarker of eosinophilic airway inflammation, remained high at 126 parts per billion.

Following prednisolone taper to <10 mg daily, the patient relapsed, with multiple presentations for upper respiratory tract symptoms and rash. Eosinophils remained undetectable, and a skin biopsy demonstrated epidermal necrosis, with neutrophilic inflammation but no vasculitis and no eosinophils. One week later, he was hospitalized with a rapidly progressive, ulcerating, purpuric rash and large joint lower limb arthralgia. His blood eosinophils were 0.6 × 10^9^/l on admission. He had a low positive perinuclear-ANCA with anti-MPO and anti-PR3 testing negative. ANA, anti-extractable nuclear antigens, anti-dsDNA antibodies, cryoglobulins and RF were negative, and complements were within normal limits. There was no active urinary sediment. His outpatient benralizumab dose had been scheduled for the day after admission but was not administered. Serum IgE was 3914 kU/l, and the blood eosinophil count rapidly escalated from 0.6 × 10^9^/l to peak at 4.6 × 10^9^/l over a 4-day period, with the first rise in blood eosinophils detected 2 weeks after the onset of the rash. A repeat skin biopsy demonstrated necrotizing vasculitis involving small and medium-sized vessels, with a dense neutrophilic infiltrate, eosinophils and granulomas.

A diagnosis of EGPA was made. His chest imaging, skin rash and histology are shown in Fig. 1. A review of his eosinophil counts, prednisolone dose and benralizumab therapies demonstrated that vasculitis progressed once CSs were weaned, while eosinophils remained undetectable. Following pulse i.v. methylprednisolone and rituximab induction for his EGPA, the patient made an excellent recovery, with clearance of the rash and perinuclear-ANCA. Blood eosinophils were eliminated with ongoing treatment with benralizumab, and the FeNO normalized after treatment of the EGPA (Supplementary Fig. S1, available at *Rheumatology Advances in Practice*). Changes in lung function are shown in Supplementary Figure S2, available at *Rheumatology Advances in Practice* online.

**Fig. 1:**
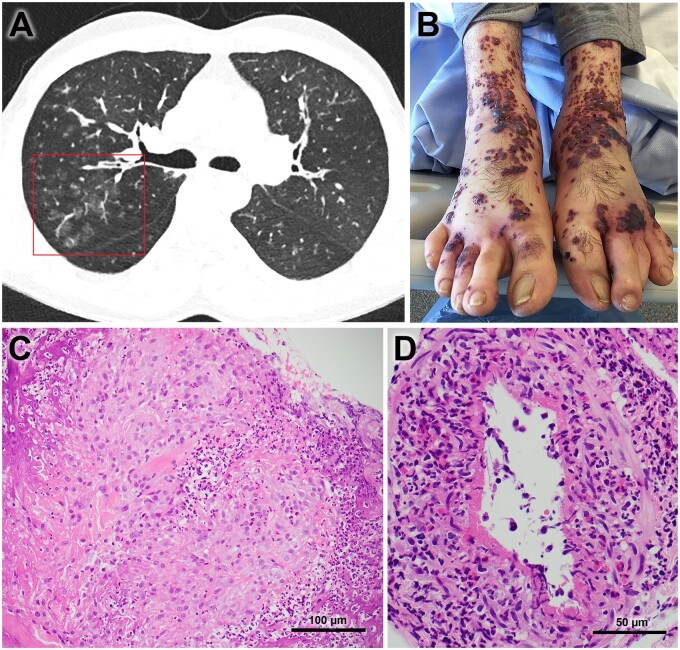
Features of eosinophilic granulomatosis with polyangiitis (**A**) Axial image from the chest CT showing patchy areas of ground-glass change and mild focal tree-in-bud nodularity. (**B**) Purpuric, papular and vesiculo-bullous rash, confluent in some areas, affecting predominantly the distal lower limbs and hands. (**C**) Skin biopsy demonstrating necrotic dermis with pyknotic cellular debris, and a large epithelioid histiocytic granuloma surrounded by an infiltrate of neutrophils (Haematoxylin and Eosin). (**D**) A dermal artery showing evidence of necrotizing vasculitis, with loss of endothelium, presence of fibrin and abundance of eosinophils (Haematoxylin and Eosin).

In this report, EGPA emerged despite depletion of circulating eosinophils by benralizumab. A previous report also described two cases of emergent EGPA on benralizumab treatment [1]. Consistent with these cases, our patient also presented with cutaneous and articular manifestations without renal involvement, while asthma control was maintained on benralizumab. In our case, blood eosinophil counts 1 week before hospitalization were able to confirm that EGPA progressed while peripheral blood eosinophils were undetectable. In common with the previous report, by the time our patient required hospitalization they had measurable eosinophils, which then increased rapidly in tandem with their cutaneous vasculitis. Given the similarities in the three cases reported, it is possible that the recrudescence of eosinophilia while on benralizumab treatment could herald the onset of the development of EGPA. Interestingly, although FeNO had remained very high despite good asthma control with maintenance oral CSs and benralizumab, it normalized after rituximab treatment. The failure to suppress FeNO in this context has not been reported before and might potentially identify a patient group with suboptimal disease control, who might be at a higher risk of developing features of vasculitis.

Benralizumab binds the IL-5 surface receptor on eosinophils, causing antibody-dependent cell-mediated cytotoxicity, usually eliminating eosinophils completely from peripheral blood. Mepolizumab (anti-IL-5) binds circulating IL-5, with less profound suppression of circulating eosinophils. Both drugs reduce exacerbations and CS requirements in severe asthma [2, 3]. Mepolizumab improves rates of remission and reduces CS requirements in EGPA [4], with similar benefits from benralizumab in EGPA suggested in several observational studies (Supplementary Table S1, available at *Rheumatology Advances in Practice*) [5–7]. A randomized placebo-controlled clinical trial is ongoing (ClinicalTrials.gov Identifier: NCT03010436).

Anti-IL-5 therapy is now widely prescribed and often permits withdrawal of maintenance CSs in severe asthma. Clinicians should be vigilant for emergence of EGPA in such cases and aware of the potential for anti-IL-5 therapy to modify classical EGPA features. EGPA tends towards two distinct patterns: MPO–ANCA-positive patients with a predominance of vasculitis features (e.g. glomerulonephritis) and ANCA-negative patients where pulmonary, sinonasal and cardiac disease are common [8]. It is conceivable that IL-5 blockade could treat the asthma and eosinophil-driven aspect of the disease without impacting on vasculitis, thereby allowing EGPA to develop despite treated asthma. Alternatively, asthma might merely represent a prodrome. Further study is required to investigate the complex relationship between anti-IL-5 therapies that might be of value in treating the disease but do not necessarily halt disease evolution.

*Funding*: No specific funding was received from any bodies in the public, commercial or not-for-profit sectors to carry out the work described in this article.

*Disclosure statement*: The authors have declared no conflicts of interest.

## Data availability statement

Data are available upon reasonable request by any qualified researchers who engage in rigorous, independent scientific research, and will be provided following review and approval of a research proposal and Statistical Analysis Plan (SAP) and execution of a Data Sharing Agreement (DSA). All data relevant to the study are included in the article.

## Supplementary data

Supplementary data are available at *Rheumatology Advances in Practice* online.

## Supplementary Material

rkab033_Supplementary_Data
