# Supplementary material for: Emergence of extrathoracic manifestations of eosinophilic granulomatosis with polyangiitis during benralizumab treatment
Source: Rheumatol Adv Pract. 2021 May 1;5(2):rkab033. doi: 10.1093/rap/rkab033 (PMC8190011; doi:10.1093/rap/rkab033)
Supplement: rkab033_Supplementary_Data [file rkab033_supplementary_data.pdf]

Supplementary Table S1. Summary of open-label observational studies of benralizumab in eosinophilic granulomatosis with polyangiitis

	Author, year [reference number]		
**Study characteristics**	**Guntur et al, 2021 [5]**	**Padoan et al, 2020 [6]**	**Nanzer et al, 2021 [7]**
Benralizumab dose [schedule]	30 mg [wk 0, 4, 8, 16, 24]	30 mg [wk 0, 4, 6, 8, 16, 24]	30 mg [wk 0, 4, 6, 8, 16, 24, 32, 40, 48]
Number of participants, n	10	5	11
Study duration, weeks	40	24	24 (n=11), 48 (n=9)
**Patient charateristics**			
Age, years	Mean 47 (SD, 17)	Median 42 (IQR, 33-56)	Mean 50 (SD, 14)
Male, n (%)	5 (50)	1 (20)	5 (45)
Disease duration, years	Mean 3.1 (SD, 4.1)	Range, 13-25 months	NR
Histological diagnosis, n (%)	6 (60)	NR	3 (27)
ANCA positive, n (%)	3 (30)	1 (20)	4 (36)
Eosinophils, cells per µL	Geometric mean 265<br>(95% CI: 81-863)	Median 1200 (IQR, 555-1495)	Median 200 (IQR, 100-700)
BVAS score	Median 10 (IQR, NR)	Median 4 (IQR, 3-4)	Mean 7.9 (SD, 3.3)
Concurrent csDMARDs, n (%)	4 (40)	5 (100), 3 failed MEP	NR, 3 failed MEP
Prednisolone dose, mg/day	Geometric mean 11.6<br>(95% CI: 6.4-20.5)	Median 12.5 (IQR, 11.25-15)	Median 15 (IQR, 10-20)
**Clinical outcomes***			
Prednisolone dose, mg/day	Geometric mean 5.3<br>(95% CI: 2.6-9.85)<br>p=0.01	Median 0 (IQR, 0-3.12)<br>p=0.01	Median 5 (IQR, 5-10) at 24 wk<br>Median 5 (IQR, 1-6.5) at 48 wk
BVAS score	No significant difference	Median 0 (IQR, 0,0)<br>p=0.01	Mean 3.45 (SD, 2.52) at 24 wk<br>p<0.001<br>Mean 3.44 (SD, 2.88) at 48 wk<br>p<0.001
Flares on benralizumab, n (%)	6 (60)<br>standardised corticosteroid taper	NR	NR
Eosinophils, cells per µL	Geometric mean 1.1<br>(95% CI: -0.2 to 4.7)<br>p<0.001	Median 0 (IQR 0,0)<br>p=0.01	Depletion in all patients

csDMARDs = conventional synthetic disease-modifying antirheumatic drugs; **MEP** = mepolizumab; **SD** = standard deviation; **IQR** = interquartile range; **BVAS** = Birmingham Vasculitis Activity Score; **NR** = not reported; **wk** = week.

*statistical significance compared to baseline values reported where available.

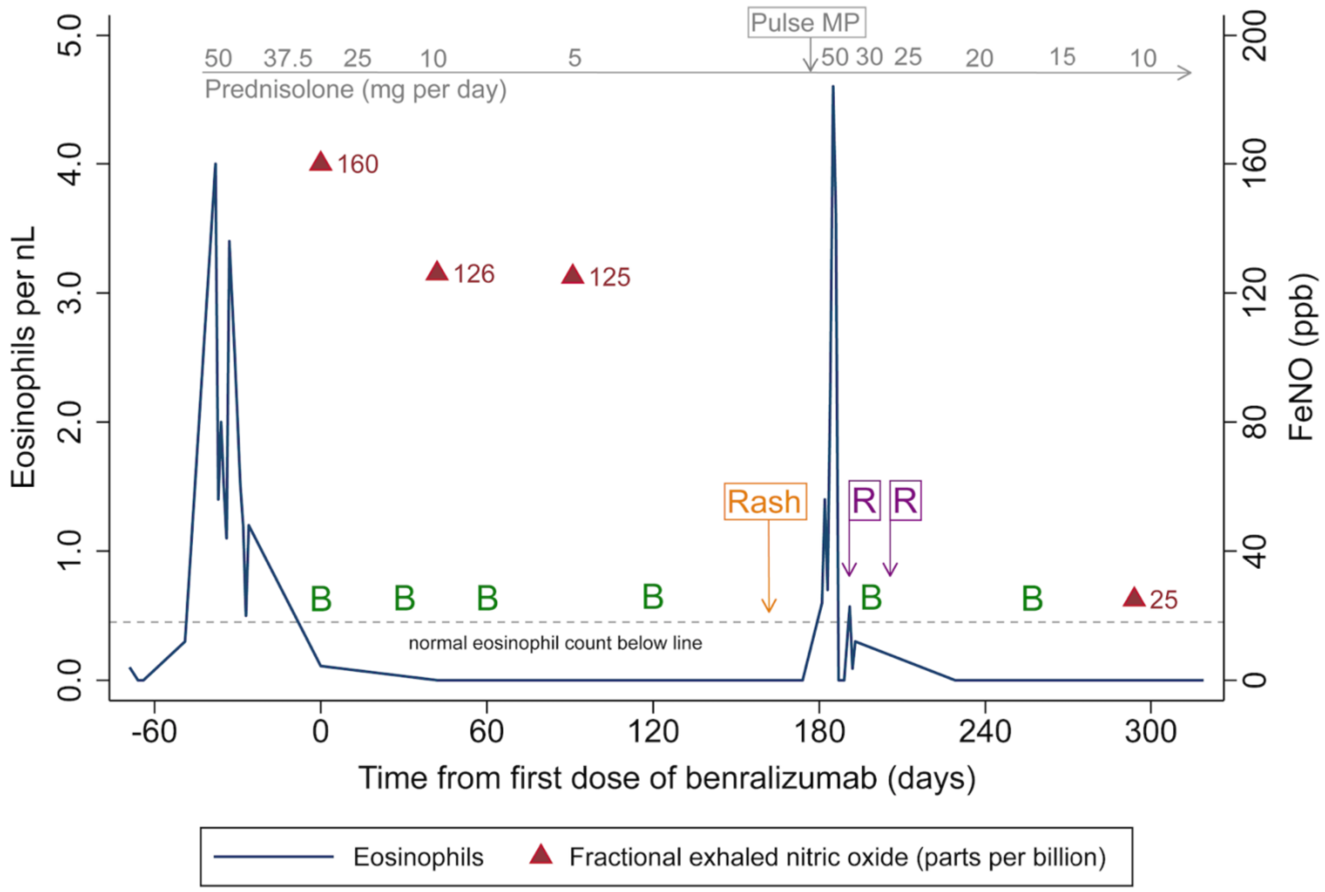

Supplementary Figure S1. Effects of treatment on blood eosinophils and fractional exhaled nitric oxide levels. After an episode of severe asthma with eosinophilia treated with oral prednisolone, the patient was initiated on benralizumab (B). His baseline fractional exhaled nitric oxide (FeNO) level was high and only declined marginally with benralizumab treatment. On steroid tapering, the patient developed a severe vasculitic rash, arthralgias and a positive p-ANCA whilst eosinophils remained undetectable. Two weeks later, eosinophil counts increased dramatically, and the patient was treated with pulse methylprednisolone (MP) and rituximab (R) induction for a diagnosis of eosinophilic granulomatosis with polyangiitis. Despite corticosteroid wean, his follow-up FeNO showed dramatic improvement after treatment of the vasculitis.

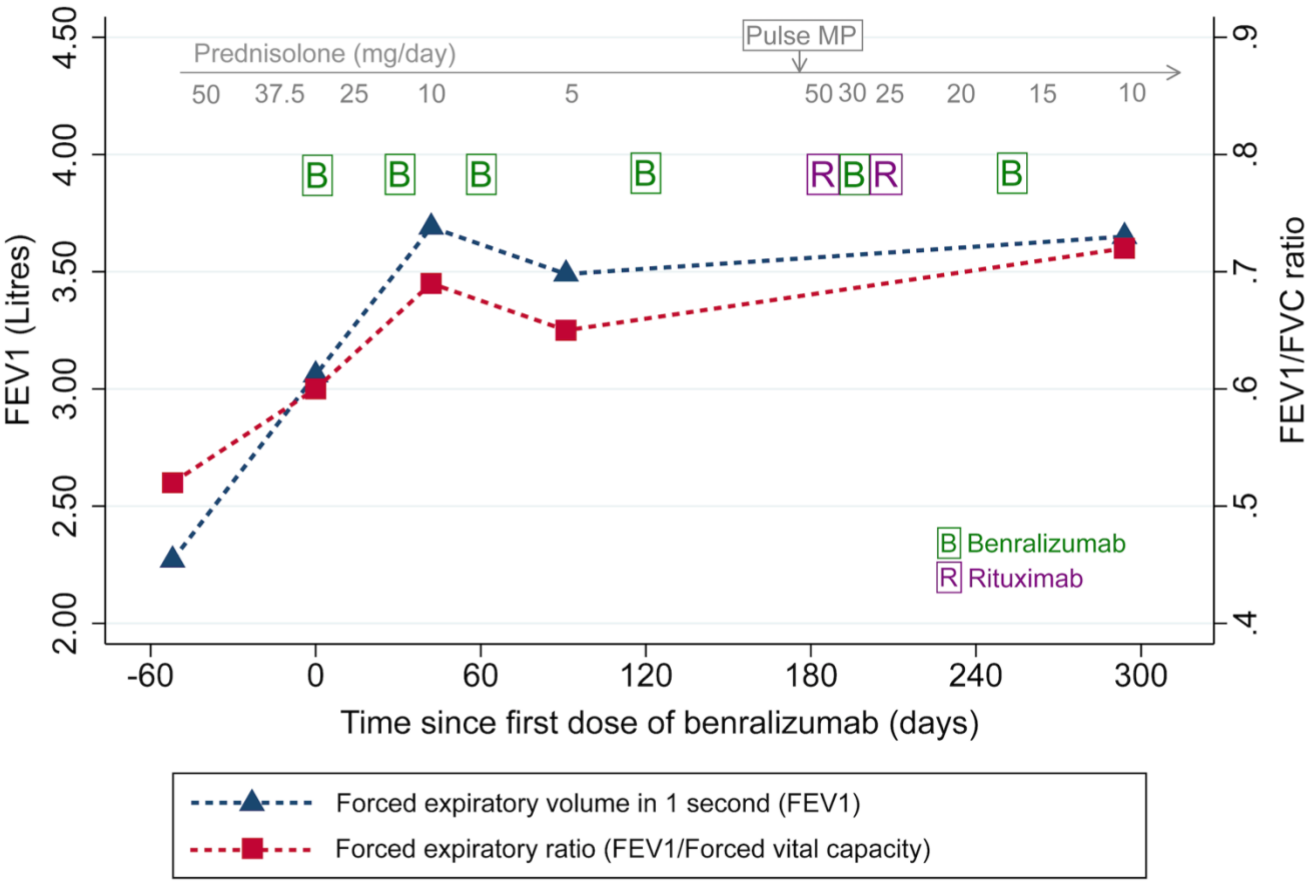

Supplementary Figure S2. Effects of treatment on serial lung function tests. At baseline, the patient had severe asthma with a post-bronchodilator forced expiratory volume in 1 second (FEV 1) of 2.27 litres (56% predicted) and a FEV 1 to force vital capacity (FVC) ratio of 0.52. With prednisolone treatment, lung function improved and was further improved by treatment with benralizumab (B) which was sustained even with corticosteroid taper. Despite effective benralizumab treatment and eosinophil depletion, the patient developed features of vasculitis and received treatment with pulse methylprednisolone (MP) and rituximab (R) induction, followed by tapering oral prednisolone. Lung function remained stable with further corticosteroid weaning.
